# Improvements in cancer survival in Hungary: a nationwide epidemiology study between 2011–2019 based on a health insurance fund database

**DOI:** 10.3389/fonc.2025.1446611

**Published:** 2025-04-03

**Authors:** Zoltán Kiss, Anikó Maráz, György Rokszin, Zsolt Horváth, Péter Nagy, Ibolya Fábián, Valéria Kovács, György Surján, Zsófia Barcza, István Kenessey, András Wéber, István Wittmann, Gergő Attila Molnár, Eszter Gyöngyösi, Viktória Buga, Miklós Darida, Tamás G. Szabó, Eugenia Karamousouli, Zsolt Abonyi-Tóth, Renáta Bertókné Tamás, Diána Fürtős, Krisztina Bogos, Judit Moldvay, Gabriella Gálffy, Lilla Tamási, Veronika Müller, Zoárd Tibor Krasznai, Gyula Ostoros, Zsolt Pápai-Székely, Gabriella Branyiczkiné Géczy, Lászlóné Hilbert, Csaba Polgár, Zoltán Vokó

**Affiliations:** ^1^ MSD Pharma Hungary Ltd., Budapest, Hungary; ^2^ Second Department of Medicine and Nephrology-Diabetes Centre, University of Pécs Medical School, Pécs, Hungary; ^3^ Department of Oncotherapy, University of Szeged, Szeged, Hungary; ^4^ RxTarget Ltd., Szolnok, Hungary; ^5^ Department of Oncology, Bács-Kiskun County Teaching Hospital, Kecskemét, Hungary; ^6^ Department of Molecular Immunology and Toxicology and the National Tumor Biology Laboratory, National Institute of Oncology, Budapest, Hungary; ^7^ Department of Anatomy and Histology, HUN-REN–UVMB Laboratory of Redox Biology Research Group, University of Veterinary Medicine, Budapest, Hungary; ^8^ Chemistry Coordinating Institute, University of Debrecen, Debrecen, Hungary; ^9^ Department of Biostatistics, University of Veterinary Medicine, Budapest, Hungary; ^10^ Department of Deputy Chief Medical Officer II., National Public Health Center, Budapest, Hungary; ^11^ Institute of Digital Health Sciences, Semmelweis University, Budapest, Hungary; ^12^ Syntesia Medical Communications Ltd., Budapest, Hungary; ^13^ Hungarian National Cancer Registry and National Tumor Biology Laboratory, National Institute of Oncology, Budapest, Hungary; ^14^ Department of Pathology, Forensic and Insurance Medicine, Semmelweis University, Budapest, Hungary; ^15^ National Korányi Institute of Pulmonology, Budapest, Hungary; ^16^ 1st Department of Pulmonology, National Korányi Institute of Pulmonology, Budapest, Hungary; ^17^ Department of Pulmonology, Albert Szent-Györgyi Medical School, University of Szeged, Szeged, Hungary; ^18^ Department of Pulmonology, Pulmonology Center of the Reformed Church in Hungary, Törökbálint, Hungary; ^19^ Department of Pulmonology, Semmelweis University, Budapest, Hungary; ^20^ Department of Obstetrics and Gynecology, Faculty of Medicine, University of Debrecen, Debrecen, Hungary; ^21^ Fejér County Szent György, University Teaching Hospital, Székesfehérvár, Hungary; ^22^ Department of Population Statistics, Hungarian Central Statistical Office, Budapest, Hungary; ^23^ National Institute of Oncology and National Tumor Biology Laboratory, Budapest, Hungary; ^24^ Department of Oncology, Semmelweis University, Budapest, Hungary; ^25^ Center for Health Technology Assessment, Semmelweis University, Budapest, Hungary; ^26^ Syreon Research Institute, Budapest, Hungary

**Keywords:** cancer, cohort study, Hungary, survival, real world data

## Abstract

**Background:**

The assessment of cancer survival is crucial for evaluating advancements in cancer management. As part of the nationwide HUN-CANCER EPI study, we examined the net survival of the Hungarian cancer patient population in 2011–2019.

**Methods:**

Using extracted data from the Hungarian National Health Insurance Fund (NHIF) database, the HUN-CANCER EPI study aimed to assess net survival probabilities for various cancer types over the past decade by the Pohar Perme Estimator method, providing insights for sex and age-specific differences and enabling comparative analysis with other European countries.

**Results:**

Between 2011 and 2019, 526,381 newly diagnosed cancer cases were identified, with colorectal, lung, breast, prostate, and bladder cancers being the most common. Age-standardized 5-year net survival rates showed significant improvements from 2011-12 till 2017-19 periods for colorectal cancer from 55.08% to 59.78% (4.70%), lung cancer from 20.10% to 23.55% (3.45%), liver cancer from 11.21% to 16.97% (5.76%) and melanoma from 90.06% to 93.80% (3.73%), while clinically relevant, but not significant improvements for breast cancer from 85.03% to 86.84% (1.81%), prostate cancer from 88.13% to 89.76% (1.63%) and thyroid cancer from 87.23% to 92.36% (5.12%). Women generally had better survival probabilities, with notable variations across cancer types. We found no significant age-related differences in cancer survival in women, while survival improvements of colorectal cancer were more pronounced in younger cohorts among male patients. International comparisons using different mortality life tables demonstrated favorable breast and prostate cancer survival rates in Hungary compared to other Central Eastern European countries.

**Conclusion:**

The HUN-CANCER EPI study revealed positive trends in cancer survival for most cancer types between 2011 and 2019. The study highlights the continued positive trajectory of cancer survival in Hungary like to more developed European countries.

## Introduction

Cancer is the second main cause of mortality after cardiovascular disease in developed countries (1). Understanding cancer survival rates and trends over time is essential to effectively improve management strategies. Moreover, generating cancer survival data serves as a crucial basis for facilitating cross-country comparisons, offering invaluable insights into the quality of a nation’s cancer management.

Overall survival is a commonly applied measure of survival rates in cancer patients which accounts for deaths from any cause. However, calculating cancer-specific survival using the concept of net survival is crucial to better understand the impact of cancer. Net survival informs us about the survival in a theoretical scenario in which cause of death could be only the disease being studied and allows survival comparisons across populations and periods of time by removing the effect of competing causes of death (2). The Pohar Perme Estimator (PPE) method estimates net survival by assuming that cancer is the only cause of death, especially for analyses with prolonged follow-up periods characterized by higher non-cancer-related mortality. Notably, it does not require individual cause-of-death data and serves as a valuable tool for comparing survival rates in different populations. Studies highlight its unbiased nature and its ability to provide consistent estimates across populations with different non-cancer mortality rates, without any identified limitations (3). A recent review identified 85 studies from 2012 to 2022 utilizing PPE for net survival estimates in cancer patients (4). However, the scarcity of such studies indicates limited use, possibly due to fewer population-based cancer studies or low awareness about this approach.

The CONCORD study group has been a consistent source for disseminating comprehensive cross-country comparisons of net survival estimates pertaining to prevalent cancer types across more than 70 countries worldwide (5,^5^). Notably, these investigations have shed light upon cancer survival disparities between post-socialist nations and their more developed counterparts (6). The evolution of net survival estimates over recent decades has been the focus of various publications, not solely confined to the work of the CONCORD study group. These collective findings underscore the evolving landscape of cancer survival rates and emphasize the need for continuous monitoring and improvement in cancer management strategies worldwide.

Survival patterns exhibit an overall positive trend, showing improvements even in the prognosis of historically more aggressive cancer types (5, 6). The International Cancer Benchmarking Partnership (ICBP) SURVMARK-2 study revealed improving survival rates for 4 out of 7 examined cancers in 7 high-income countries, however, it showed persistent international disparities which may be explained by differences in disease stage at diagnosis, access to treatment, and the presence of comorbidities (7).

Hungary has never been included in comprehensive cross-country net survival estimates, nor has any such publication or analysis been reported from our country despite the availability of a National Cancer Registry and a comprehensive National Health Insurance Fund (NHIF) database, both of which offer robust foundations for such research endeavors (8–13). Furthermore, the cross-country comparison of survival estimates heavily relies on the methodology used for calculating net survival (14).

Therefore, the primary objective of the HUN-CANCER EPI study was to assess net survival for all relevant cancer types over the past decade with a diverse methodology. Our aim was to provide crucial information concerning age- and sex-specific differences, and to present a comprehensive picture of the changes in these estimates over a decade-long period. Lastly, we sought to compare our findings with those from other European countries.

## Materials and methods

### Study design

This is a nationwide, retrospective study designed to evaluate cancer incidence and outcomes among the Hungarian population.

Patient Recruitment: Patients were included if they were newly diagnosed with any type of cancer (ICD-10 codes: C00–97, excluding C44) between January 1, 2011, and December 31, 2019. A screening period from 2009 to 2010 was applied to exclude prevalent cancer cases.

### Data sources

Our study utilized the databases of the Hungarian National Health Insurance Fund (NHIF) and the Hungarian Central Statistical Office (HCSO). The NHIF database encompasses almost the entire Hungarian population, including details on drug prescriptions, hospital admissions, outpatient consultations, and medical interventions. It also contains medical information related to diagnostic codes, according to the International Statistical Classification of Diseases and Related Health Problems 10^th^ Revision (ICD-10) (15).

### Variables collected

From the NHIF and HCSO databases, the following variables were extracted: Unique personal social security number (for record linkage), dates of cancer diagnosis and death, ICD-10 diagnostic codes and their frequency (to determine the dominant tumor type in cases of multiple diagnoses), details on drug prescriptions, hospital admissions, outpatient consultations, and medical interventions, additional clinical and administrative data used for sensitivity analyses and validation of cancer definitions (see **Supplementary Table 1**).

Our current analysis focused on patients diagnosed with any type of cancer (ICD-10 codes: C00–97, excluding C44) between January 1, 2011, and December 31, 2019. The identification of records from different sources was based on unique personal social security numbers. To calculate annual cancer incidence rates, the NHIF database was queried for individuals having a cancer-related ICD-10 code in at least two distinct reimbursement records. The fact and date of death are regularly updated by the National Health Insurance Fund based on data from the State Population Registry Office. Patients who died within 60 days of the first reported ICD-10 code of interest were also included. If a patient had two or more different cancer-related ICD-10 codes, the ICD-10 code group with a higher number of associated occurrences was considered. For instance, if both the breast cancer-related ICD-10 code C50 and the lung cancer-related C34 code appeared in the reports, but more reimbursement entries were related to C50, the patient was classified as having breast cancer. This approach helped to exclude coding mistakes (e.g., metastasis of breast cancer in the lung coded as primary lung cancer, as the NHIF database is not a medical registry but a reimbursement-focused database). The date of diagnosis was defined as the first appearance of the identified cancer-related ICD-10 code. However, second or multiple primary malignancies were ruled out from further analysis, the implications of which are detailed in the limitation section. When defining the ‘dominant’ tumor type in patients with multiple cancer types, only ICD-10 codes with at least two occurrences were considered. To allow for international comparisons, we clustered patients into the following groups in line with Ferlay’s publications (5, 16). Patients who did not have any of the described ICD-10 codes described by Ferlay et al. but had an ICD-10 code starting with C (except for C44) were classified as having other cancer. ICD-10 codes of C44 (non-melanoma skin cancer) were excluded in accordance with international cancer epidemiology studies.

A screening period was set from 2009 to 2010 to exclude patients with prevalent cancers and accurately identify newly diagnosed cancer patients from 2011 onward. To test the sensitivity of our definitions in the query, we carried out multiple calculations based on different cancer-related treatment patterns of patients with cancer and measured the accuracy of cancer definitions (**Supplementary Table 1**). A detailed description of sensitivity analyses validating the accuracy of tumor type definitions was reported in our previous publication (17).

### Statistical analysis

For comparability with the CONCORD-3 and other recent studies, we calculated 5-year net survival for cancer types according to Ferlay’s classification, applying the Pohar Perme methodology (18). Subsequently, raw net survival estimates were age-standardized based on the methodology described by Corazziari et al. in 2004 (19). We estimated net survival using the Pohar-Perme estimator, which is widely regarded as a robust method in population-based cancer studies, as it corrects for the effects of competing mortality. The essence of the Pohar-Perme method is to determine the excess mortality rate for each patient (i-th patient) on each day of the study period (j-th day) using the following formula:


$$\lambda_{j}=\frac{\Sigma_{i}w_{ij}d_{ij}-\Sigma_{i}w_{ij}d_{ij}^{*}}{\Sigma_{i}w_{ij}y_{ij}}$$

where *y_ij_* =1 if the i patient was still alive up to day j; *d_ij_* = 1 if the patient i died on day j, otherwise 0; $d_{ij}^{*}$ : the population hazard for patient i; *w_ij_*: the reciprocal of the expected survival of patient i up to day j. Using the formula above, we calculate the cumulative hazard as follows: $\Lambda_{j}=\Sigma_{j}\lambda_{j}$ ; and the net survival: $S_{j}^{*}=\exp(-\Lambda_{j})$ .For age-standardized net survival estimates, we applied the age-group weights recommended by the International Cancer Survival Standard (ICSS) and calculated as follows: $\sum_{k=1}^{5}w_{k}S_{k}^{*}$ , where *w_k_* is the weight for the k-th age group and $S_{k}^{*}$ is the net survival in the k-th age group. This approach enables meaningful comparison of survival rates across populations by mitigating age-related biases (20). The analysis was conducted for the diagnostic periods 2011–2012, 2013–2014, 2015–2016, 2017–2018, 2017–2019, 2011–2014 and 2015–2019.

For our study, we utilized both the Human Life-Table Database (HLD) and the Hungarian Mortality Database (HMD) as background period mortality tables, each serving a distinct purpose in our net survival analysis (21). The HLD was employed for Pohar-Perme estimates, providing mortality data up to age 100. Patients were followed until September 30, 2022, at which point censoring occurred. Specifically, patients were followed until death if they passed away before age 100 or were censored at this age, aligning with the HLD’s upper age limit. To ensure the accuracy of our data, we supplemented the HLD with updated information from the HCSO up to 2022. This approach allowed us to incorporate the most current mortality rates available.

Additionally, to enhance cross-country comparability, net survival outcomes were calculated using the HMD as well. The HMD provides mortality data with an extended right-censoring point, up to age 110, and includes data until the end of 2020, with follow-up extending to December 31, 2020. This broader age range offered by the HMD allows for alternative survival estimates that may better reflect differences in longevity across populations.

The Pohar-Perme method supports the use of both mortality databases, allowing us to capture nuanced survival trends in Hungary and make our findings more comparable internationally. These dual sources enabled us to provide comprehensive and reliable net survival estimates tailored to the Hungarian context, while also aligning with global standards for comparative analysis.

For standardization according to the methodology outlined by Corazziari et al. in 2004, raw net survival rates were computed for the age groups 15–44, 45–54, 55–64, 65–74, and 75–99 years, except for prostate cancer, where the age groups 15–54, 55–64, 65–74, 75–84, and 85–99 were considered, and patients aged 100 or older were excluded from net survival analyses. Although the publication in question specified separate age groupings for bone cancers, bone cancers do not constitute a distinct group according to Ferlay’s classification, therefore, such categorization was not applied. In line with Corazziari et al., no gender correction was implemented and the age standardized net survival was calculated as the weighted mean of the net survival derived for the individual age groups. To apply the appropriate International Cancer Survival Standard (ICSS) weights, we categorized Ferlay’s cancer types into one of three standard populations based on the age distribution of the cancers: (i) increasing incidence with age, (ii) approximately constant incidence with age, and (iii) cancers predominantly affecting young adults. For certain cancer types, clear categorization was challenging due to the inclusion of diagnoses falling into different standards. In such cases, the respective cancer type was classified into standard 1 (see further details in **Supplementary Table 2**).

For better cross-country comparisons, net survival outcomes were also calculated using the HMD (URL: https://www.mortality.org/), which provides data up to the age of 110, until the year 2020, with the end of the follow-up period being December 31, 2020. The Pohar Perme method supports the use of both mortality databases (18).

In terms of certain tumor characteristics, we also examined the trends in net survival by age group. For this analysis, we utilized raw net survival rates instead of age-standardized values. The analysis was conducted across cohorts defined by the following age ranges: 0-19, 20-29, 30-39, 40-49, 50-59, 60-69, 70-79, and 80+.

All calculations detailed above, were carried out in R v4.0.4 (2021-02-15) using relsurv package (Available from: https://www.r-project.org).

We conducted a trend analysis of 5-year net survival rates using multidimensional penalized splines (MPSs) as described by Dantony et al. (22). This approach models the dynamic excess mortality hazard across time since diagnosis, age at diagnosis, and year of diagnosis, providing flexibility to capture complex survival patterns while avoiding overfitting through penalization. The R package survPen was employed for this analysis, enabling us to evaluate survival trends across multiple cancer types between 2011 and 2018. Results were supplemented with Pohar-Perme net survival estimates for validation and comparison. All analyses were performed using anonymized national cancer registry data, ensuring comprehensive and robust evaluation of survival trends.

The study was approved by the National Ethical Committee (IV/298-2/2022/EKU).

## Results

We identified a total of 526,381 newly diagnosed cancer cases in the NHIF database between 2011 and 2019, with a nearly equal distribution of males (49.43%, n=260,206) and females (50.57%, n=266,175). The most commonly diagnosed cancers were lung, colorectal, breast, prostate, and bladder cancers, reflecting known patterns of cancer prevalence in Hungary (**Supplementary Table 3**).

### Progress in age-standardized 5-year net survival rates

**Figure 1:**
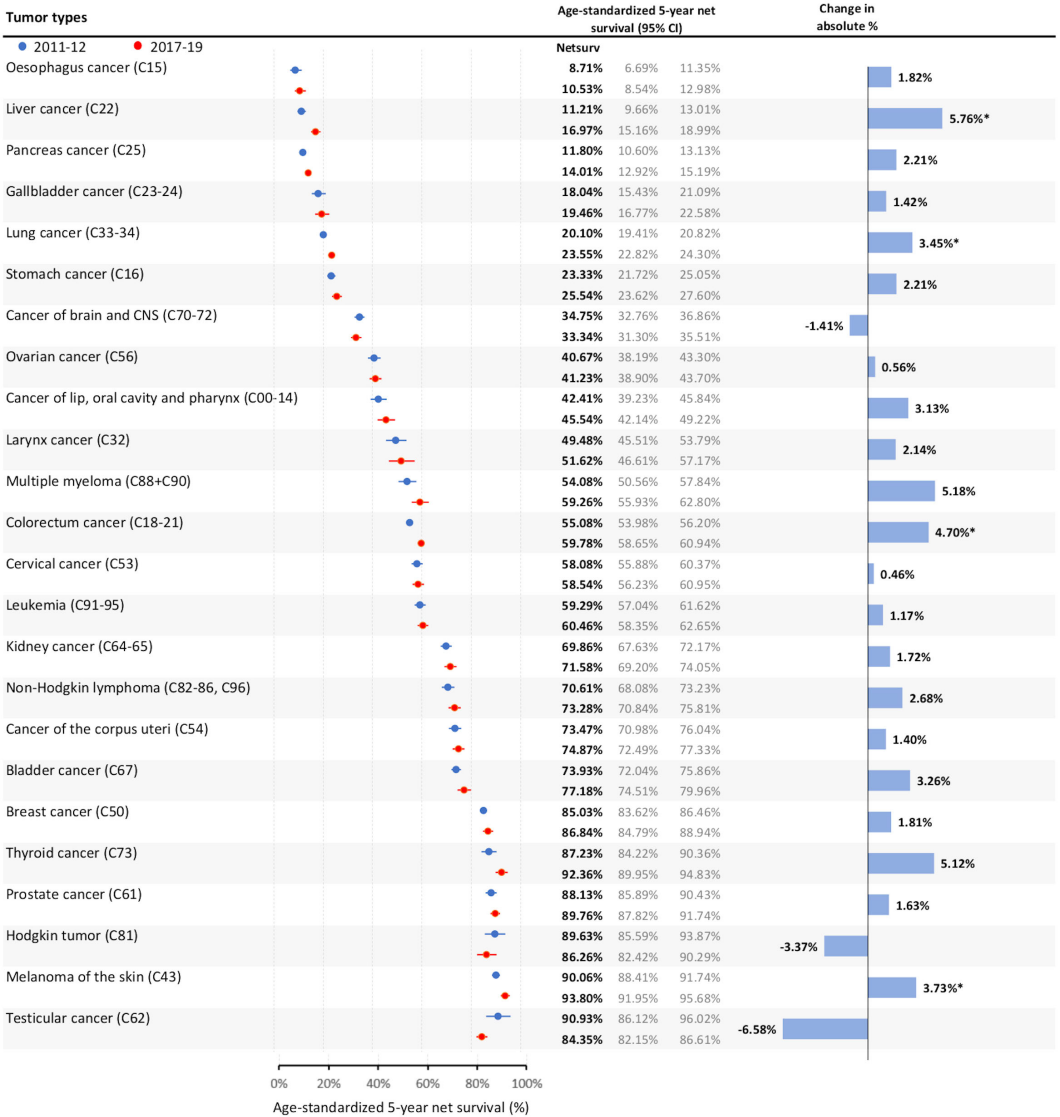
Age-standardized 5-year net survival of Hungarian cancer patients diagnosed in 2011–12 vs. 2017–19 by tumor type and the absolute percentage change in net survival. (HLD mortality life table – December 31, 2022) *p<0.05.

Our analysis reveals meaningful progress in 5-year net survival rates for various cancers, highlighting both improvements and areas requiring further attention (**Figure 1**; **Supplementary Table 4** - using HDL). For colorectal cancer, we observed a substantial increase in survival from 55.08% (95%CI: 53.98%-56.20%) in 2011–2012 to 59.78% (95%CI: 58.65%-60.94%) in 2017–2019, an absolute rise of 4.7%. Breast cancer survival saw a modest increase from 85.03% to 86.84%, though this 1.81% change was not statistically significant. Lung cancer, historically challenging to treat due to late-stage diagnosis, showed a notable survival gain from 20.10% to 23.55% (absolute increase: 3.45%, significant). Liver cancer and melanoma showed the highest significant improvements in survival at 5.76% and 3.73%, respectively, highlighting areas where interventions may have particularly strengthened outcomes. Thyroid cancer, while not statistically significant, saw a 5.12% increase in survival, underscoring advances in the management of cancers with relatively high baseline survival. However, for certain cancers like brain tumors, Hodgkin lymphoma, and testicular cancer, we noted slight declines in 5-year survival (e.g., -1.41%, -3.37%, and -6.58%, respectively), though these were not statistically significant, warranting further investigation into potential disparities in these areas.

### Gender-specific trends in survival

**Supplementary Figures 1A, B** provide a gender-based breakdown, revealing distinct survival trends among males and females. For males, significant increases were observed in stomach cancer (up 5.91%) and melanoma (up 6.52%), suggesting potential improvements in male-specific cancer prevention and management efforts. Colorectal and lung cancers in males also demonstrated meaningful gains, with respective increases of 5.78% and 3.49%. Conversely, a slight but noticeable decline was observed in cancers like Hodgkin lymphoma (-6.53%) and gallbladder cancer (-6.08%), emphasizing the need for targeted strategies in male cancer survival.

Among females, survival improved significantly for liver cancer (8.72%), lung cancer (2.83%), colorectal cancer (3.57%), and thyroid cancer (6.62%), and may reflect potential advances in treatment accessibility and response among women. The observed gender differences, particularly the survival improvements in colorectal and liver cancers, suggest that gender-specific factors, including biology, health-seeking behaviors, or differential access to care, may influence outcomes.

### Sex-related differences in 5-year net survival

**Figure 2:**
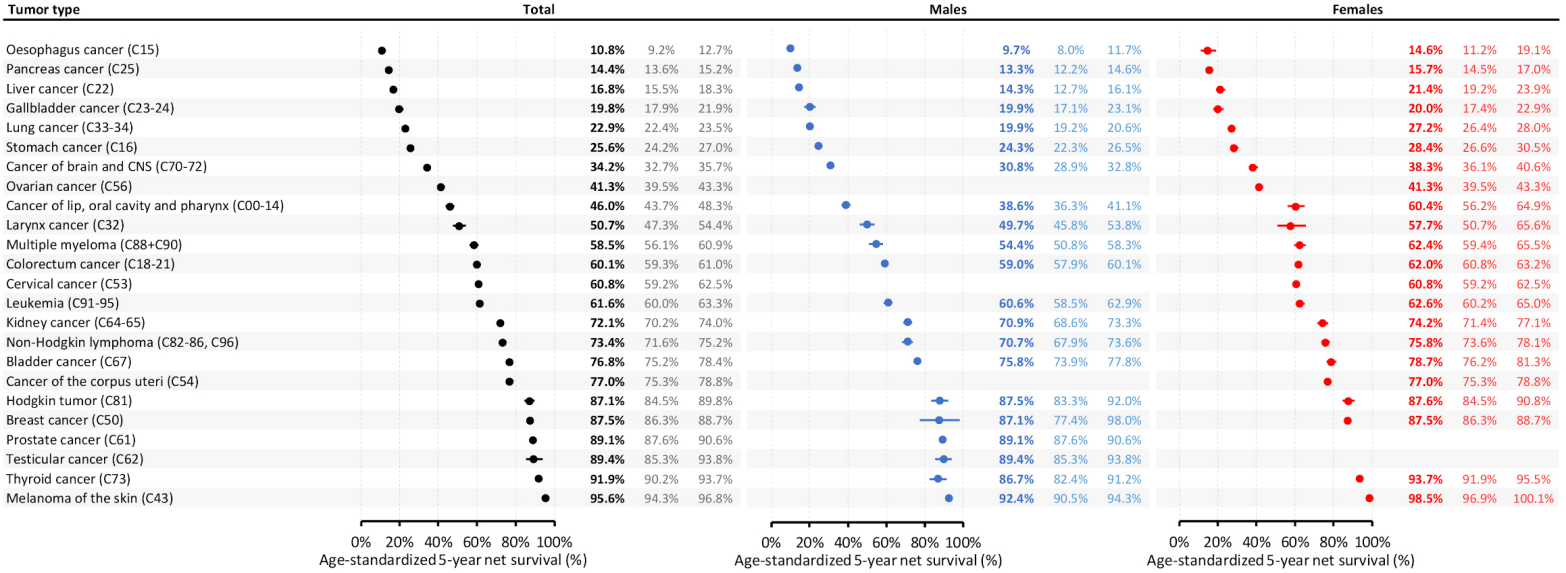
Age-standardized 5-year net survival of Hungarian cancer patients diagnosed between 2015 and 2019 by different tumor types and sex (HLD mortality life table – December 31, 2022).

Our analysis using the HLD mortality life table highlights that females generally exhibit higher net survival rates across most cancer types compared to males, as shown in **Figure 2**. For cancers diagnosed between 2015 and 2019, females demonstrated notable survival advantages: lung cancer survival was 7.3% higher in females (as absolute difference; 27.2% vs. 19.9% in males), and laryngeal cancer showed an 8.0% survival advantage for females (as absolute difference; 57.7% vs. 49.7%). Similarly, females had better survival outcomes for melanoma (98.5% vs. 92.4% in males, a 6.1% advantage), liver cancer (7.1% absolute difference), multiple myeloma (8.0%), and thyroid cancer (7.0%). The most pronounced sex-based difference was seen in cancers of the lip, oral cavity, and pharynx, where females had a 21.7% higher survival rate than males. Colorectal cancer also demonstrated a gender survival gap, with females showing a 62.0% 5-year net survival rate compared to 59.0% in males for those diagnosed between 2015 and 2019. These results suggest possible underlying biological, behavioral, or healthcare-access factors that may benefit females more than males in certain cancer types. Detailed sex-related differences for earlier diagnostic periods are shown in **Supplementary Figure 2A**, with additional international comparisons using the Pohar Perme method with HMD life tables in **Supplementary Figures 2B, C**.

### Age- and sex-related differences in 5-year net survival

**Figure 3:**
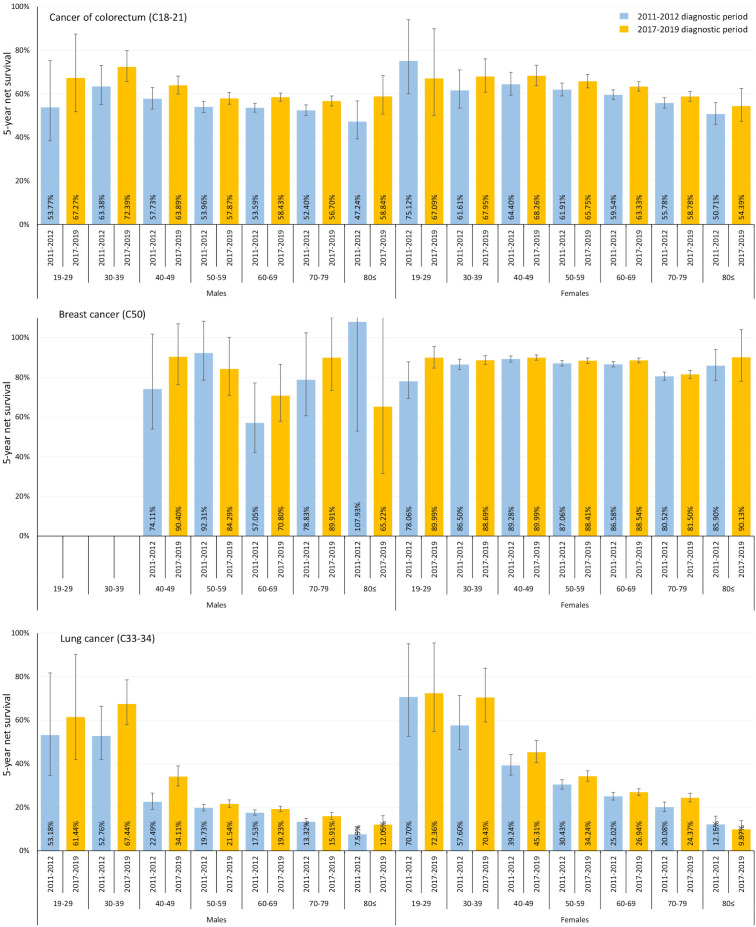
Five-year net survival of patients with colorectal cancer (C18-21), breast cancer (C50) and lung cancer (C33-34) by age and sex in Hungary during the 2011–2012 and 2017–2019 periods. (HLD mortality life table – December 31, 2022).

Although females had better survival rates in general, males showed more pronounced survival improvements during the study period. We did not find any significant age-related differences in the net survival of female patients with colorectal cancer (**Figure 3**). On the other hand, among male colorectal cancer patients, 5-year net survival showed more pronounced improvements between the 2011–2012 and 2017–2019 diagnostic periods in younger age cohorts, than in older ones. Similarly to colorectal cancer, 5-year net survival of breast cancer did not show any significant age-related differences among females, neither in the 2011–2012, nor in the 2017–2019 diagnostic period. However, for lung cancer, younger cohorts had much better 5-year net survival, than the older population. Age-related net survival results at different timepoints (1, 3, and 5 years) for all cancer types and for all study periods are shown in **Supplementary Table 4** using the HLD mortality life table and in **Supplementary Table 5** using the HMD mortality life table.

### Hungarian age-standardized 5-year net survival estimates compared to the CONCORD-3 study and international results

**Figure 4:**
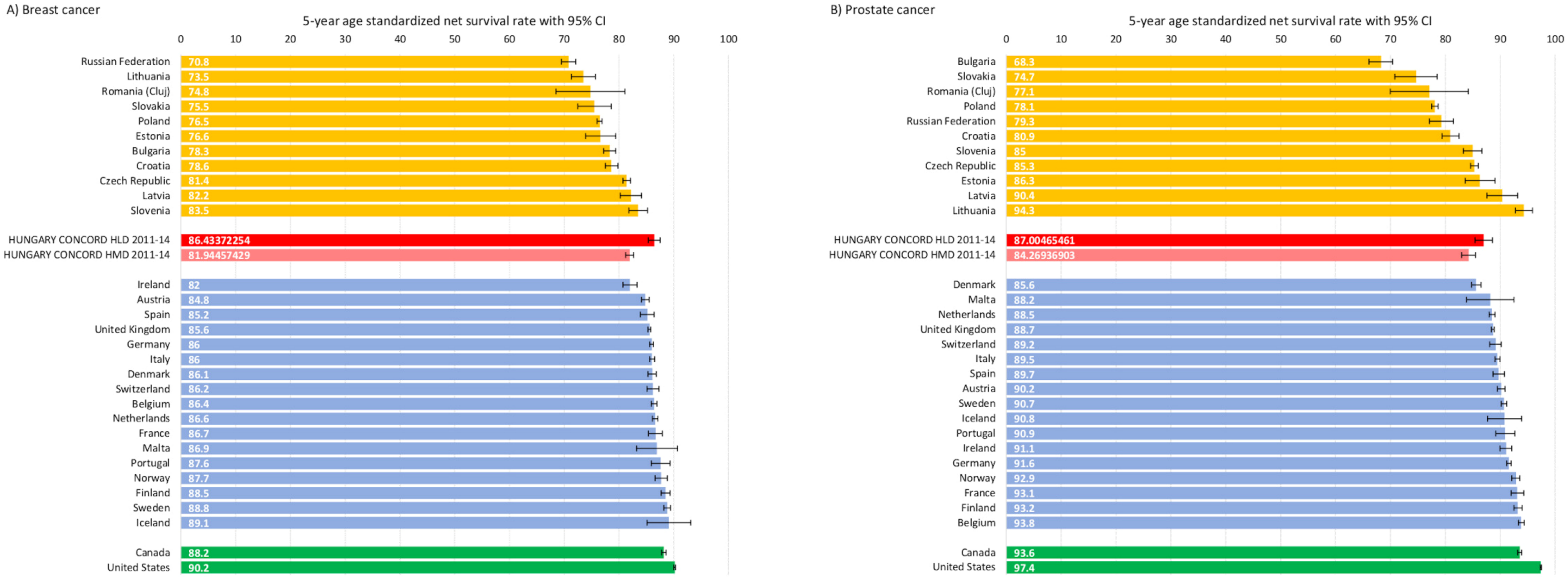
Age-standardized 5-year net survival of breast cancer **(A)** and prostate cancer **(B)** in Hungary (red bars) (with HLD and HMD life tables) for patients diagnosed during the 2011–2014 period compared to CONCORD-3 study group results for Europe (yellow bars for Eastern-Europe, blue for Western-Europe) and U.S. and Canada (green bars) during the 2010-2014 period.

As net survival calculations may differ according to the mortality life tables applied (HLD vs. HMD), we calculated 5-year net survival using the Pohar Perme method with both mortality life tables to allow for comparability with international results (**Supplementary Tables 3**, **4**). HLD and HMD results of age standardized 5-year net survival of Hungarian breast cancer patients (diagnosed in 2011–2014) were compared to the CONCORD-3 study group European results for the 2010–2014 period (**Figure 4A**). We found a 5-year net survival of 86.4% for this tumor type with HLD, and 81.9% with HMD, which seem to be among the highest in the Central Eastern European (CEE) region (Czech Republic: 81.4%; Slovenia 83.5%), and comparable to results from more developed countries. **Figure 4B** shows the age-standardized 5-year net survival rates of Hungarian prostate cancer patients diagnosed in the 2011–2014 period (HLD: 87.0%), which also seem to be among the highest in the region. However, Norway, France, Finland, Belgium, the United States (U.S.), and Canada showed much better outcomes for roughly the same diagnostic period.

### Survival trends across cancer types

**Figure 5:**
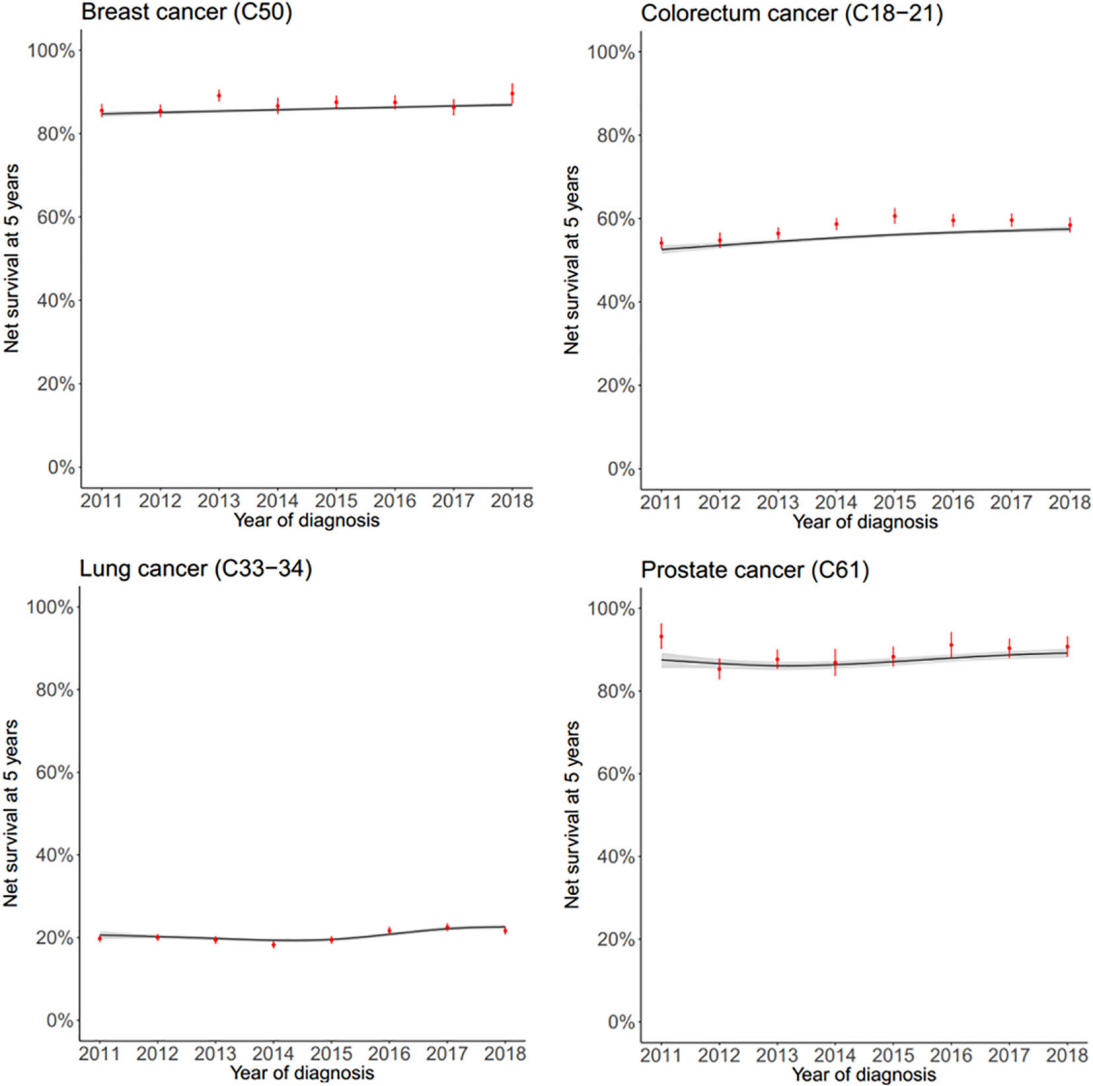
Year-by-year tabulation of net survival rates of breast cancer, colorectal cancer, lung cancer and prostate cancer (both sex) of the calculated MPS-derived trends and using Pohar-Perme method.

The trend analysis using multidimensional penalized splines (MPSs) revealed significant improvements in 5-year net survival rates for several cancer types between 2011 and 2018. Marked survival gains were observed for breast cancer, head and neck cancers, colorectal cancer, gallbladder cancer, kidney cancer, leukemia, multiple myeloma, liver cancer, melanoma, ovarian cancer, and thyroid cancer. For these cancers, the survival trends consistently showed an upward trajectory over the study period. For other cancer types, no clear directional trend was observed, indicating stable survival rates during this timeframe. The results are visually summarized in **Figure 5**; **Supplementary Figure 3**, which include both MPS-derived survival estimates and Pohar-Perme 5-year net survival rates, each presented with 95% confidence intervals. Additionally, the supplementary tables provide year-by-year trend estimates and corresponding Pohar-Perme results for a comprehensive understanding of the data (**Supplementary Table 6**, **7**). These findings highlight the varying dynamics of cancer survival trends in Hungary, underscoring progress in certain areas and the need for further investigation in others.

## Discussion

Our nationwide retrospective study was conducted as part of the Hungarian (HUN-CANCER EPI) Cancer Epidemiology program and aimed to evaluate net survival rates of cancer in a Central Eastern European country. Our objective was to provide a comprehensive overview of net survival trends, providing the first analysis of its kind from a CEE nation by including nearly all cancer cases diagnosed between 2011 and 2019. We conducted analyses assessing both short-term (1–3 years) and long-term (5 years) survival rates across various cancer types, as well as according to age and sex. Our most significant findings were the favorable trends in survival rates across nearly all cancer types throughout the study period. Additionally, we performed net survival analyses employing the Pohar Perme method while utilizing two different mortality life tables. This approach aimed to establish a foundation for cross-country comparisons encompassing various methods for estimating net survival.

In most developed countries, cancer survival rates have significantly improved due to advancements in diagnosis, treatment modalities, and a better understanding of the disease’s molecular mechanisms (7, 23). The CONCORD study group reported notable positive trends in cancer survival for various cancer types, including those historically considered more aggressive (5). The SURVMARK-2 project analyzed 3.9 million cancer cases in 7 high-income countries and showed increases in survival rates between 1995 and 2014 for most cancer types, with younger patients having greater improvements (7). However, the study also revealed cross-country differences in survival. A Slovenian cancer registry recently reported an 11% increase in 5-year net cancer survival during a 20-year study period. Male patients showed more significant improvements, and age and stage at diagnosis were significantly associated with survival (23). A population-based study of the Spanish Network of Cancer Registries (REDECAN) analyzed over 600,000 adult cancer cases and found an increase in survival rates from 2002–2007 to 2008-2013, particularly for colorectal cancer and cancers usually associated with poor prognosis (24). SURVCAN-3, an international collaboration of population-based cancer registries, found significant disparities related to the level of development of a country as well as the availability and effectiveness of healthcare services (25).

### Development of age-standardized 5-year net survival in Hungary in the light of international data

In the HUN-CANCPER EPI study, we also found relevant increases in net survival for most cancer types during the 2011–2019 period. The highest, approximately 5% improvements were detected for colorectal cancer, multiple myeloma, thyroid cancer, liver cancer and melanoma. Improvements were statistically significant for colorectal cancer (4.70%), liver cancer (5.76%), melanoma (3.73%) and lung cancer (3.45%), partly due to the high number of diagnosed patients. Male patients showed more pronounced and significant survival improvements for melanoma (6.52%), stomach cancer (5.91%), and colorectal cancer (5.78%), and clinically relevant improvements for kidney cancer, bladder cancers, multiple myeloma, and liver cancer. In females, the most significant increases were seen for head and neck cancers (10.02%), thyroid cancer (6.62%), and liver cancer (8.72%). Improvements in cancer survival in Hungary is in line with previous international findings mentioned above (7, 23, 24). However, the magnitude of improvement is not directly comparable due to the different study periods examined in these studies. Our study shows that survival improvements observed in the 1990s and 2000s continued in the 2010s in Hungary.

The additional trend analysis using multidimensional penalized splines (MPSs) provided deeper insights into survival improvements. This method confirmed and refined the findings of the direct period comparisons, particularly for breast cancer, head and neck cancers, colorectal cancer, liver cancer, and melanoma, where marked survival gains were consistently observed. For some cancers, such as gallbladder and ovarian cancers, the trend analysis revealed a more pronounced improvement over time, which was not as evident in the simple comparison of the 2011–2012 and 2017–2019 periods. Conversely, cancers with no directional trend, such as lung and bladder cancers, exhibited stable survival rates, underscoring areas where further advancements are needed.

The advanced methodology of MPSs enabled a more transparent and accurate evaluation of survival trends, mitigating potential biases introduced by discrete period comparisons. For instance, the simple evaluation of 2017–2019 survival data could be confounded by the emerging effects of the COVID-19 pandemic, which disproportionately affected healthcare systems and cancer outcomes. In contrast, the trend analysis method provides a robust framework for distinguishing genuine survival improvements from external disruptions. These findings highlight the potential of advanced statistical approaches to more precisely assess progress in cancer control efforts and identify gaps for targeted interventions.

### Hungarian net survival of different cancer types in aspect of international results

The first period of the HUN-CANCER EPI study coincided with certain analyses from the CONCORD-3 and SURVMARK-2 studies as well as with survival analyses from Slovenia These analyses all applied the Pohar Perme method, although we need to emphasize that using different mortality life tables may lead to different net survival results for certain cancer types generally associated with longer survival and older patient populations. For example, using the Human Mortality Database (age cohorts 0–110 years), we found 5-year net survival rates of 81.9%, 84.3%, 54.8% and 89.4% for breast cancer, prostate cancer, colorectal cancer, and melanoma, respectively, while the application of the Human Life-Table Database (age cohorts 0–99 years) resulted in survival rates of 86.4%, 87.0%, 56.6% and 91.8% for the same cancer types, respectively.

The CONCORD-3 study group reported 5-year prostate cancer net survival rates of 68.3–94.3% for CEE countries, and 85.6–93.8% for Western European (WE) countries for the 2010–2014 period (5). Our study shows that the net survival of Hungarian prostate cancer patients was high among CEE countries but did not reach that of WE countries during the same period. Slovenian analyses reported 92.3% net survival for the 2012–2016 period, which is higher than the Hungarian results. However, the Slovenian rate reported by the CONCORD-3 study was 85% for 2010–2014, demonstrating that the analyses are highly sensitive to the mortality life tables used. In our study, 5-year net survival of breast cancer was found to be 81.9% using the HMD table and 86.4% using the HLD table, both of which are higher than rates reported by the CONCORD-3 study group for CEE countries, and comparable to WE countries. This may be attributed to higher participation on screening, modern diagnostic opportunities, and comparable access to modern therapies. For colorectal cancer, we found 5-year net survival rates of 54.8% (HMD) and 56.6% (HLD) in 2010–2014, which is lower but comparable to those reported by the CONCORD-3 study for CEE countries (e.g. Slovenia: 61.7%). Our results for melanoma and lung cancer were also comparable with findings from Slovenia and from the CONCORD-3 study for CEE countries both with the use of HMD and HLD. On the other hand, 5-year net survival of cervical cancer was in the middle range among CEE countries and much lower than in WE countries, highlighting a significant unmet need. In summary, Hungary was one of the better performers in the CEE region in terms of cancer survival, albeit with still poorer outcomes compared to WE. Of note, the past few years have witnessed a narrowing in the cancer survival gap between CEE and WE countries, although reports from the end of the 2010s are still scarce (5, 16). It is important to emphasize again that cross-country net survival comparisons should be interpreted with caution due to differences in study methodology and the quality of data reporting (5).

### Age- and sex-related differences in 5-year net survival

In our study, female cancer patients had better net survival compared to male patients for most cancer types during the same periods. Sex-related differences in net survival were around 7% in favor of women for laryngeal and lung cancer and around 5% for liver cancer, thyroid cancer, multiple myeloma, and melanoma in 2015–2019. The survival advantage of female cancer patients is well-documented (26, 27) and may be attributed to tumor characteristics and differences in risk factors such as hormone levels, infections, and chromosomal alterations. Of note, smoking is a major risk factor associated with cancer mortality (28, 29). In the Hungarian adult population, smoking is significantly more prevalent among men, than in women (30), which has a profound impact on cancer survival. Recently, there has been a decrease in smoking prevalence among men (opposite to the increase in women), which may explain the more pronounced cancer survival improvements seen in male patients in our study. Previous studies also suggest that men have a higher comorbidity burden at the time of cancer diagnosis compared to women (31, 32), which may also influence net survival rates. We also found age-related differences in cancer survival for various cancer types. Of note, younger patients with melanoma, breast, colorectal, thyroid, and prostate cancer tended to have fairly similar net survival probabilities compared to older cohorts. However, significant differences were found for lung, stomach, cervical, kidney, ovarian, and pancreatic cancer, with older cohorts showing worse survival rates. The SURVMARK-2 study showed clear survival improvements among younger patients (<75 years) with more aggressive tumor types which was attributed to their relatively broader access to adjuvant chemotherapy, better tolerance for more aggressive treatment regimens (24). Net survival analyses from Slovenia reported higher and more pronounced improvements in survival rates for patients aged 20–49 years, with a 15% increase from 1997–2011 to 2012–2016. On the other hand, patients older than 75 years had the lowest survival rates despite a 7% improvement over the past 20 years (7). Despite therapeutic advancements, the effective treatment of elderly cancer patients remains challenging due to common side effects and comorbidities.

Less pronounced age-related differences were found in net survival for cancers with higher public awareness and media coverage as well as effective screening programs such as breast cancer, prostate cancer, and melanoma. Patients with breast cancer had very similar survival probabilities irrespective of age, which can further be explained by differences in the prevalence of breast cancer subtypes across age groups (33, 34).

### Strengths and limitations

A key strength of our study lies in the substantial number of cancer patients identified during the study period, increasing the statistical reliability of our findings. Rigorous data cleaning procedures were implemented to ensure accuracy and validity. Moreover, the extensive decade-long follow-up period provided a broad perspective on cancer trends over time. The nationwide nature of the NHIF database allowed for a more comprehensive evaluation of cancer outcomes in the country. Additionally, our methodology involved the incorporation of cancer-related interventions, which allowed for the exclusion of cases with incorrectly applied cancer-associated ICD codes which did not align with the patient’s condition or outcome.

However, there are certain limitations. Our methodology relied on cancer-related ICD code records, potentially excluding patients with secondary or multiple primary tumors and resulting in an underestimation of cancer incidence. Our 9-year retrospective database analysis might not have captured cases where patients initially diagnosed with one primary tumor developed another type of primary cancer during the follow-up period, impacting the interpretation of results and understanding changes in cancer diagnoses over time. In previous studies, multiple primary neoplasms accounted for around 3% percentage of cancers within a 5-year long period (35–37), which would correspond to around 1,500 missed new cancer cases every year in our study. Notably, our data lacked detailed information on molecular histology, TNM stage, and ECOG status, limiting our ability to assess survival rates by specific subtypes and examine the influence of patient-related factors on cancer survival probability. We must emphasize that during the 2017-2019 diagnostic period, not all diagnosed patients had 5-year long survival which population was censored from the net-survival estimation. This censoring nevertheless was non-informative (i.e., it did not depend on the future prognosis of these patients conditional on being alive at the time of censoring). Therefore, it was unlikely to bias our results. However, as the survival probability depended on the time period studied, thus the survival estimates for this period and closer to the time point of 5 years, might have been somewhat underestimated, as these estimates are based on the data solely of patients diagnosed earlier. Besides, the Covid-19 pandemic may also have impact on the 5-year net survival of those cancer patients, whom were diagnosed between 2017-2019.

Although this study focused on two key diagnostic periods (2011-2012 and 2017-2019) to provide an overall perspective on cancer survival trends, future studies could benefit from a detailed trend analysis across annual intervals. Such analysis may leverage flexible methodologies that can accommodate the unique, often non-linear nature of survival data. We are currently developing a longitudinal study that will analyze net survival trends for cancer patients diagnosed between 2011 and 2024, which will allow for a more nuanced trend evaluation once the full dataset becomes available.

## Conclusion

The nationwide Hungarian (HUN-CANCER EPI) Cancer Epidemiology study revealed positive trends in cancer survival rates during the 2011–2019 period. Notably, improvements were observed for colorectal cancer, multiple myeloma, thyroid cancer, liver cancer, and melanoma. Hungary has shown a continued positive trajectory in cancer survival, similarly to more developed European countries. Females generally showed better survival rates, which may be explained by the higher prevalence of smoking among men in Hungary. Age differences in survival vary across cancer types, demonstrating the complex interplay between age, stage at diagnosis, and treatment outcomes. The findings highlight the evolving landscape of cancer survival in Hungary, calling for targeted interventions and further research.

## Data Availability

The original contributions presented in the study are included in the article/**Supplementary Material**. Further inquiries can be directed to the corresponding author.

## Supplementary material

Supplementary Figure 1
**(A)** Age-standardized 5-year net survival of Hungarian male cancer patients diagnosed in 2011–2012 and 2017–2019 by tumor type, and the absolute percentage change of net survival. (HLD mortality life table – December 31, 2022) **(B)** Age-standardized 5-year net survival of Hungarian female cancer patients diagnosed in 2011–2012 and 2017–2019 by different tumor type, and the absolute percentage change of net survival. (HLD mortality life table – December 31, 2022)

Supplementary Figure 2
**(A)** Age-standardized 5-year net survival of Hungarian cancer patients diagnosed in 2011–2014 by sex and tumor type (HLD mortality life table – December 31, 2022) **(B)** Age-standardized 5-year net survival of Hungarian cancer patients diagnosed in 2015–2019 by sex and tumor type and (HMD mortality life table – December 31, 2020) **(C)** Age-standardized 5-year net survival of Hungarian cancer patients diagnosed in 2011–2014 by sex and tumor type (HMD mortality life table – December 31, 2020).

Supplementary Figure 3 Year-by-year tabulation of net survival rates of breast cancer, colorectal cancer, lung cancer and prostate cancer (both sex) of the calculated MPS-derived trends and using Pohar-Perme method.

Supplementary Table 1 Sensitivity analyses of cancer type definitions by different cancer interventions.

Supplementary Table 2 Standard populations based on the age distribution of the cancers: (1) increasing incidence with age, (2) approximately constant incidence with age, and (3) cancers predominantly affecting.

Supplementary Table 3 Number of diagnosed Hungarian cancer patients by different cancer types (between 2011 and 2019).

Supplementary Table 4 1-, 3- and 5-year net survival of Hungarian cancer patients diagnosed between 2011–2019 by sex, age cohorts and tumor type (HLD mortality table – December 31, 2022).
