# Supplementary figures and images for: Improvements in cancer survival in Hungary: a nationwide epidemiology study between 2011–2019 based on a health insurance fund database
Source: Front Oncol. 2025 Apr 3;15:1446611. doi: 10.3389/fonc.2025.1446611 (PMC12004281; doi:10.3389/fonc.2025.1446611)

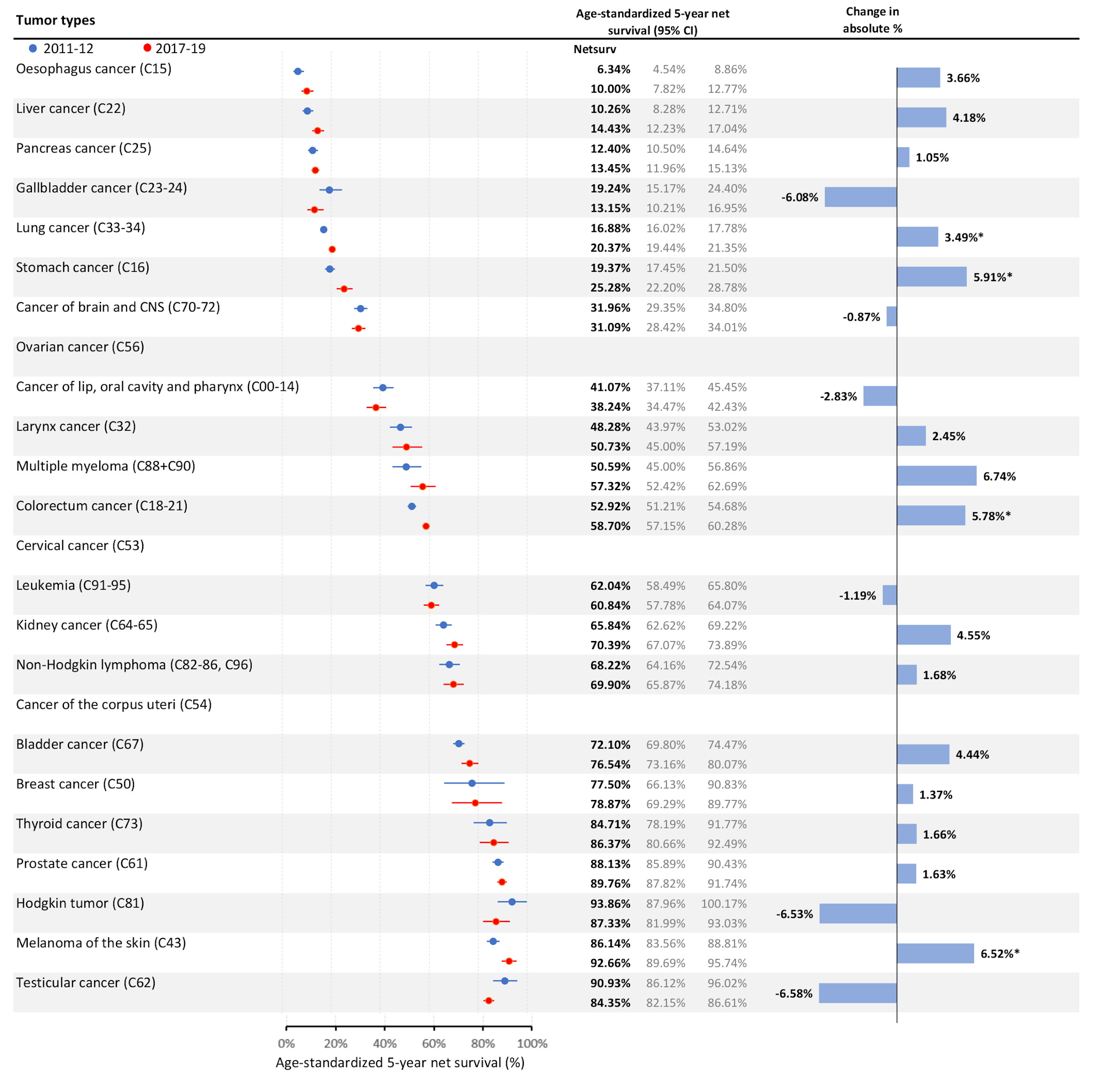

Supplement: Supplementary Figure 1 — (A) Age-standardized 5-year net survival of Hungarian male cancer patients diagnosed in 2011–2012 and 2017–2019 by tumor type, and the absolute percentage change of net survival. (HLD mortality life table – December 31, 2022) (B) Age-standardized 5-year net survival of Hungarian female cancer patients diagnosed in 2011–2012 and 2017–2019 by different tumor type, and the absolute percentage change of net survival. (HLD mortality life table – December 31, 2022) [file Image1.jpeg]

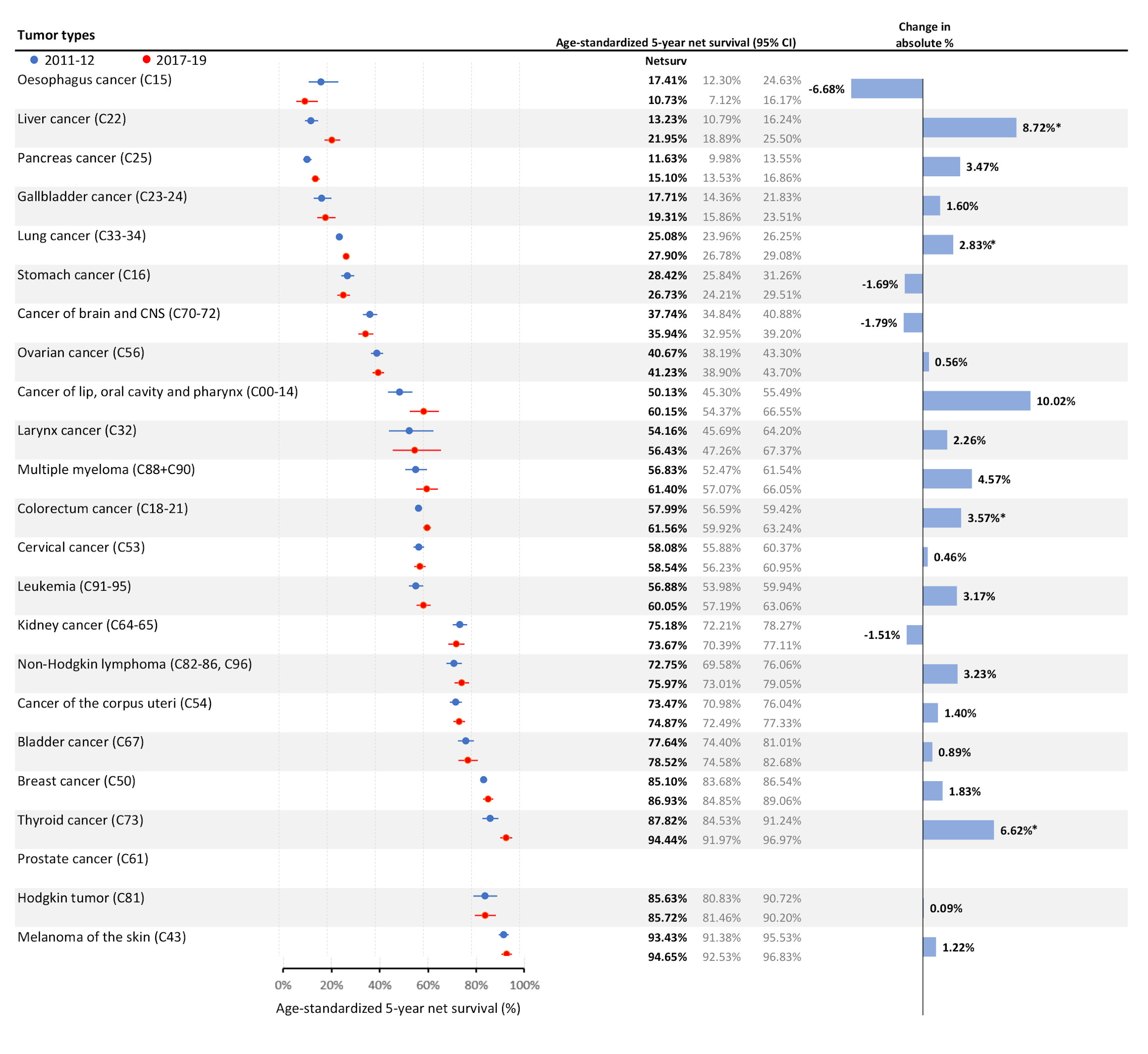

Supplement: Supplementary Figure 2 — (A) Age-standardized 5-year net survival of Hungarian cancer patients diagnosed in 2011–2014 by sex and tumor type (HLD mortality life table – December 31, 2022) (B) Age-standardized 5-year net survival of Hungarian cancer patients diagnosed in 2015–2019 by sex and tumor type and (HMD mortality life table – December 31, 2020) (C) Age-standardized 5-year net survival of Hungarian cancer patients diagnosed in 2011–2014 by sex and tumor type (HMD mortality life table – December 31, 2020). [file Image2.jpeg]

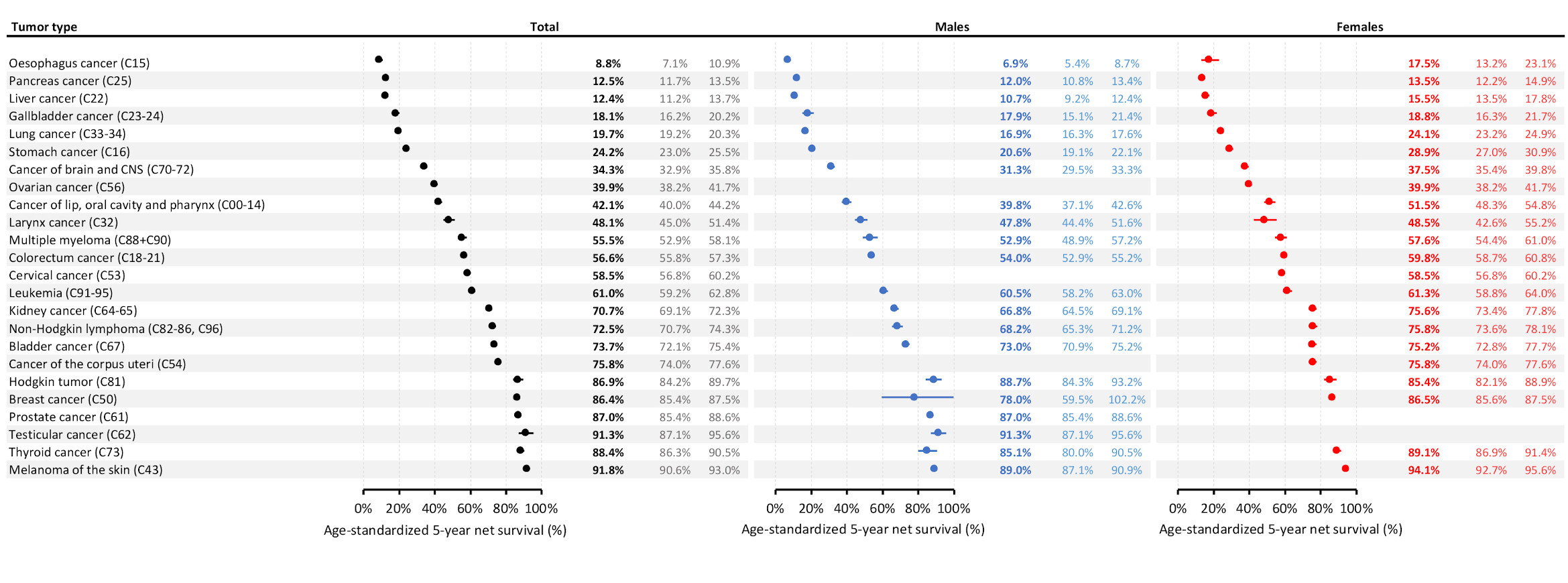

Supplement: Supplementary Figure 3 — Year-by-year tabulation of net survival rates of breast cancer, colorectal cancer, lung cancer and prostate cancer (both sex) of the calculated MPS-derived trends and using Pohar-Perme method. [file Image3.jpeg]

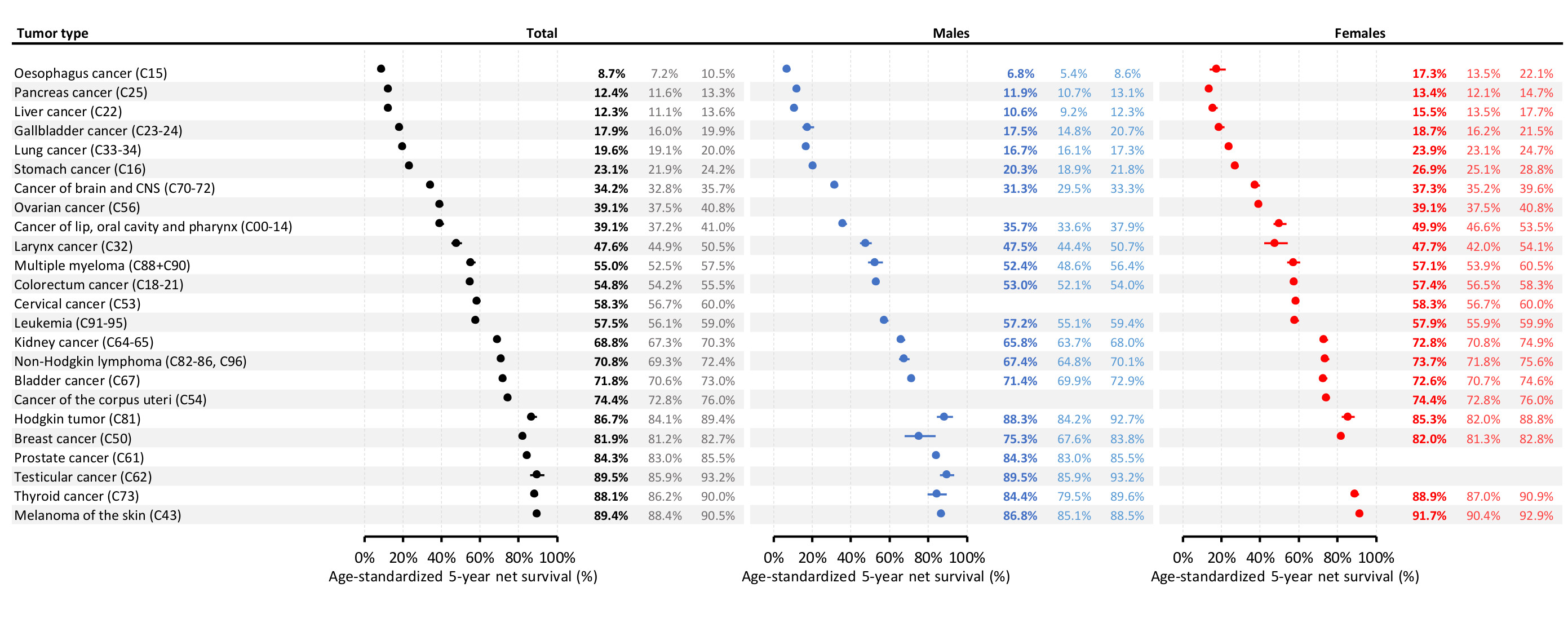

Supplement: Supplementary file 5 [file Image4.jpeg]

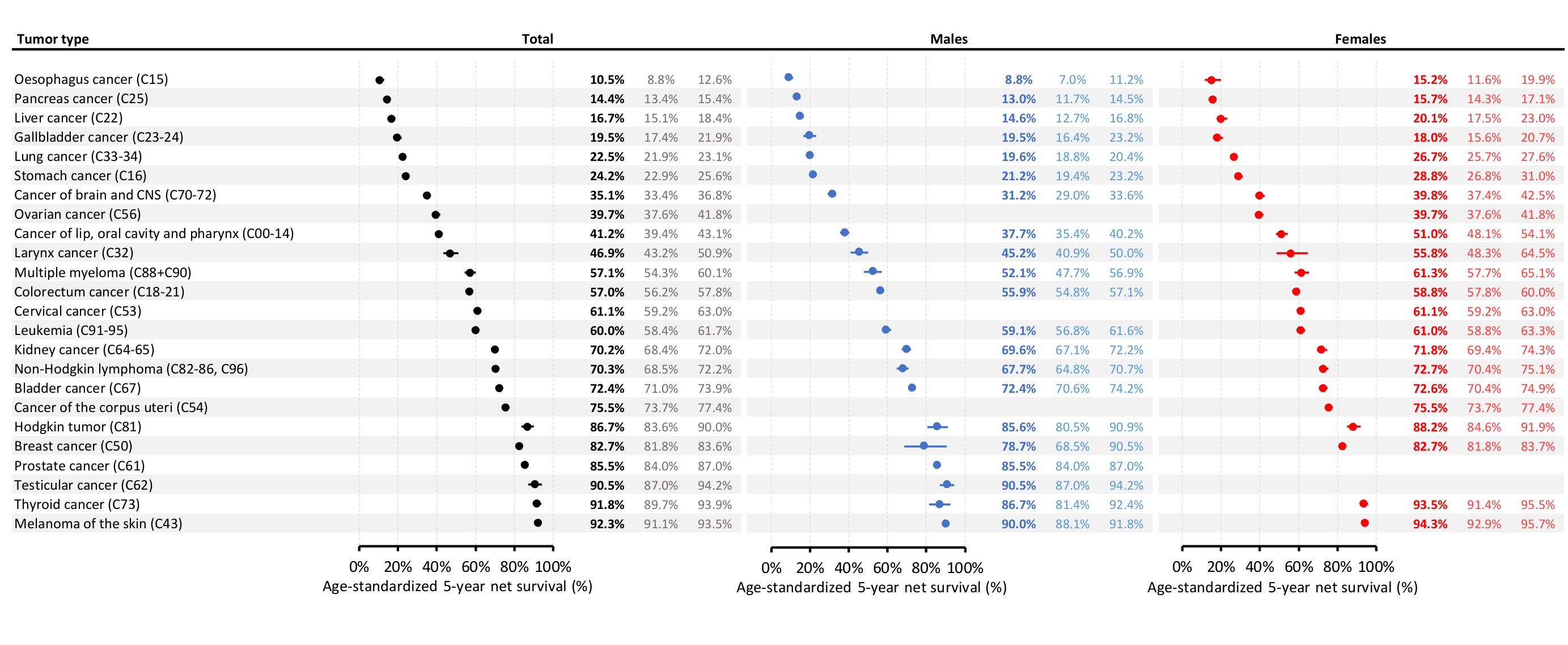

Supplement: Supplementary file 6 [file Image5.jpeg]

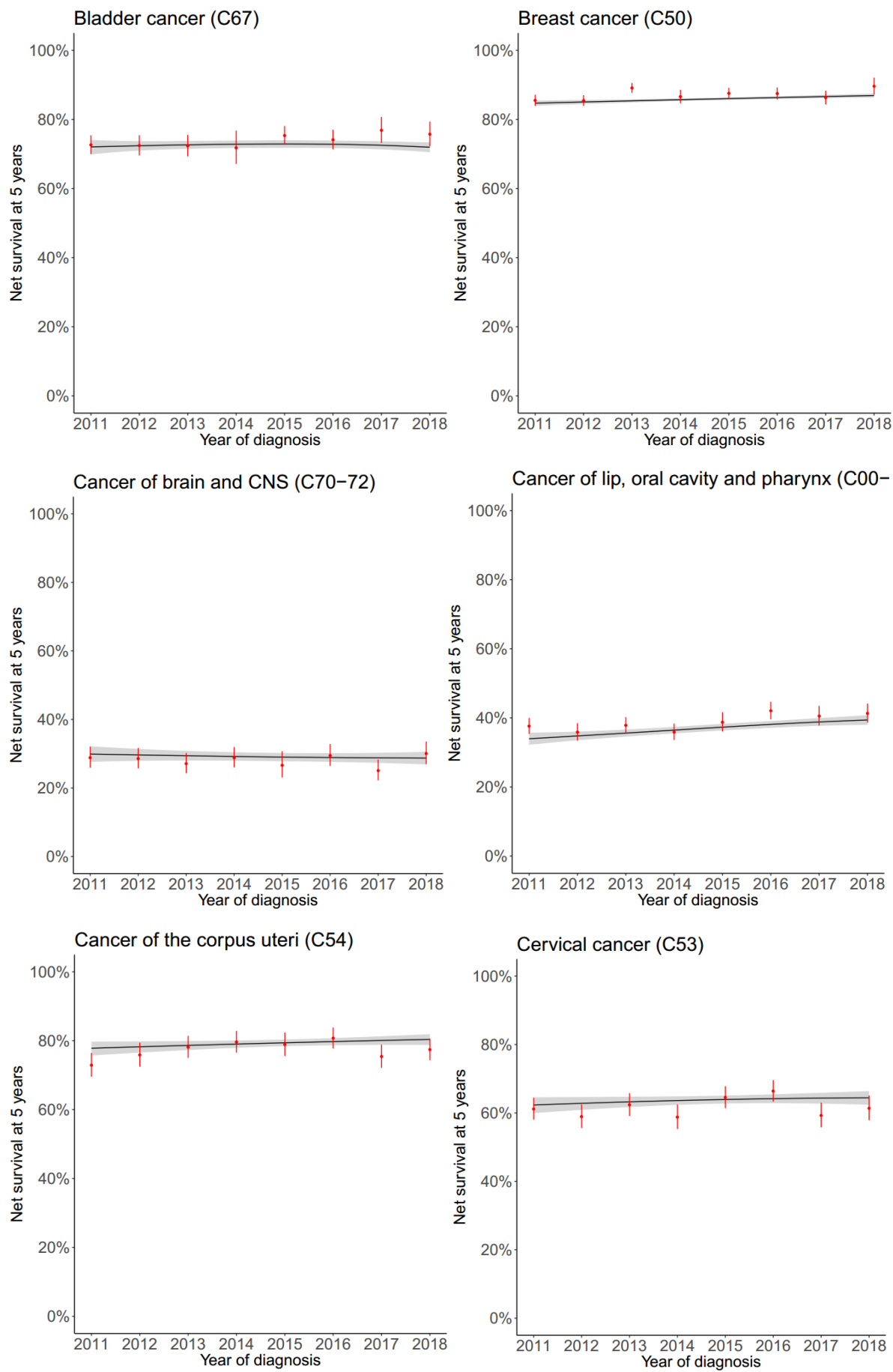

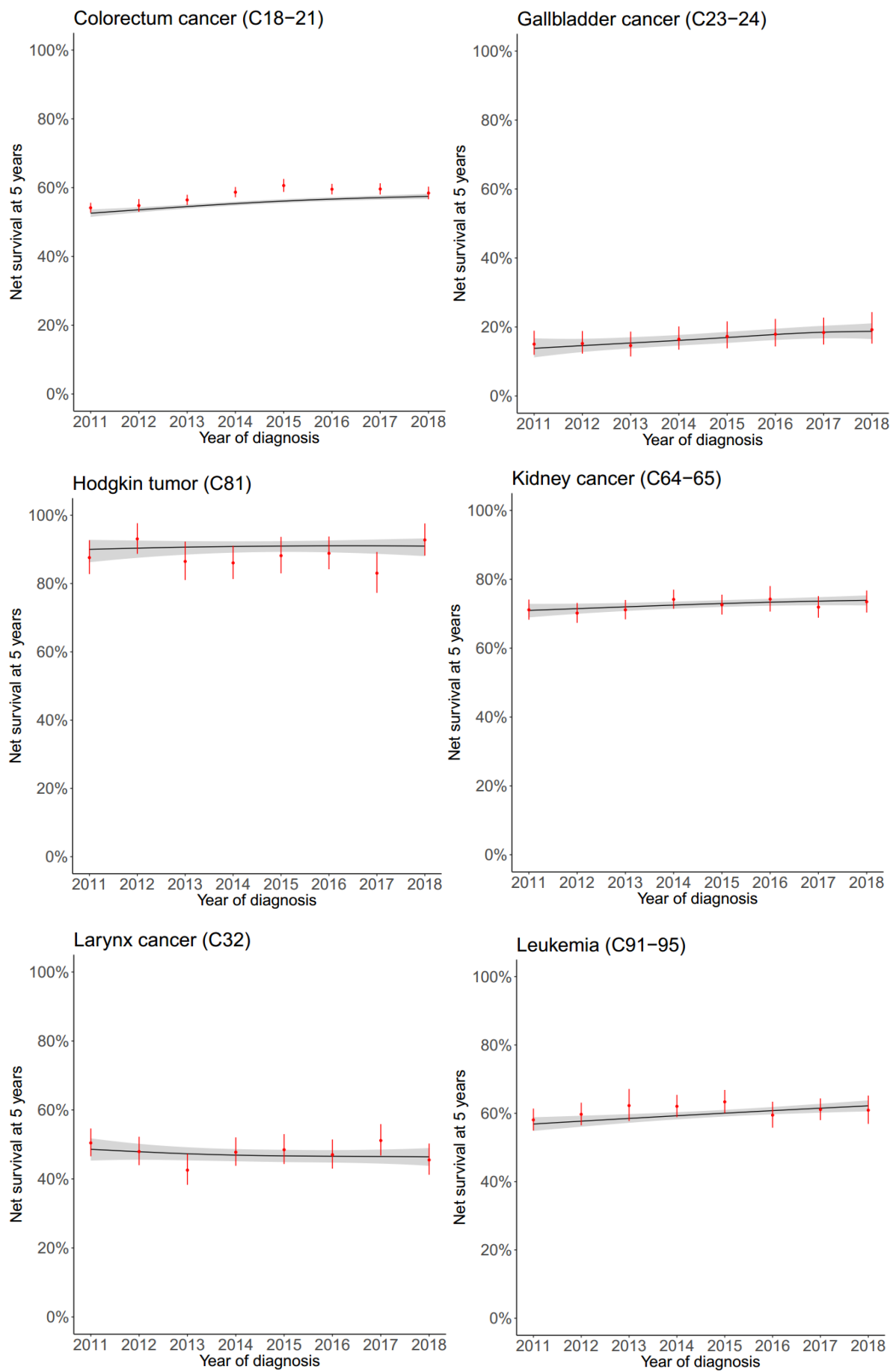

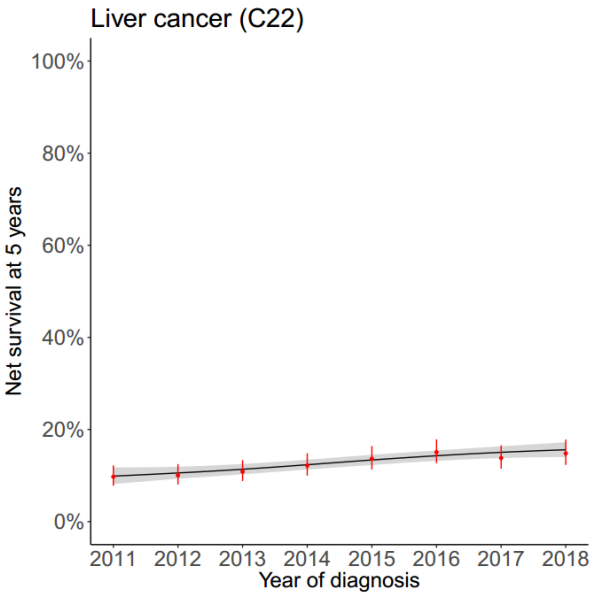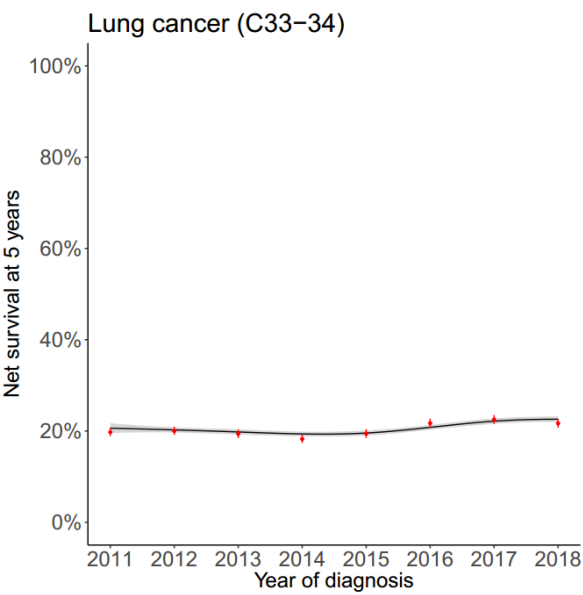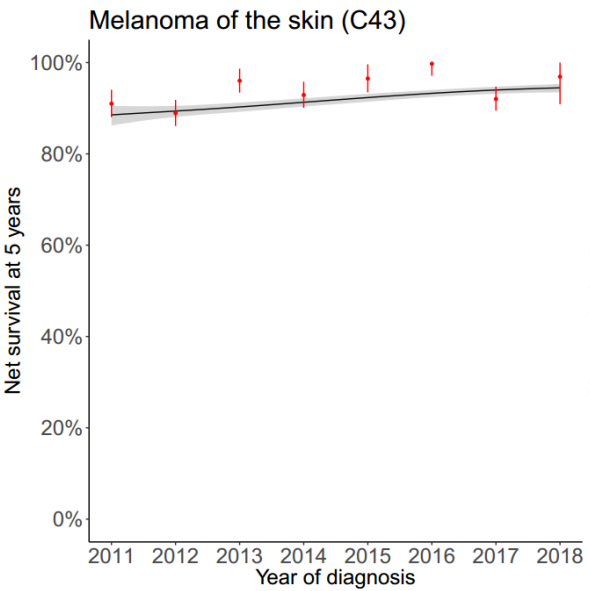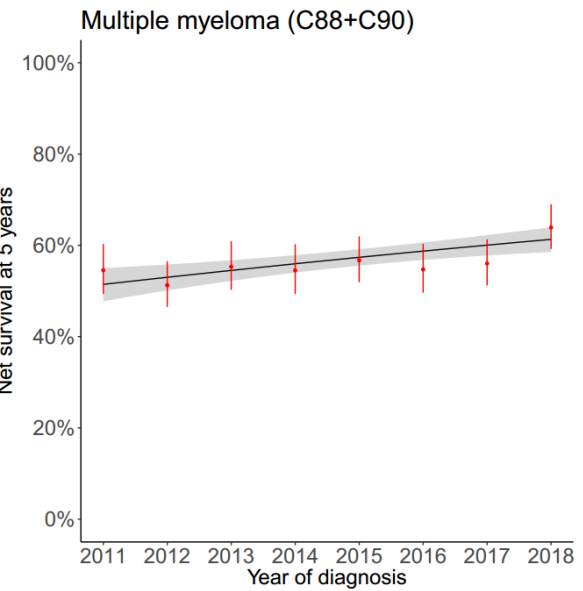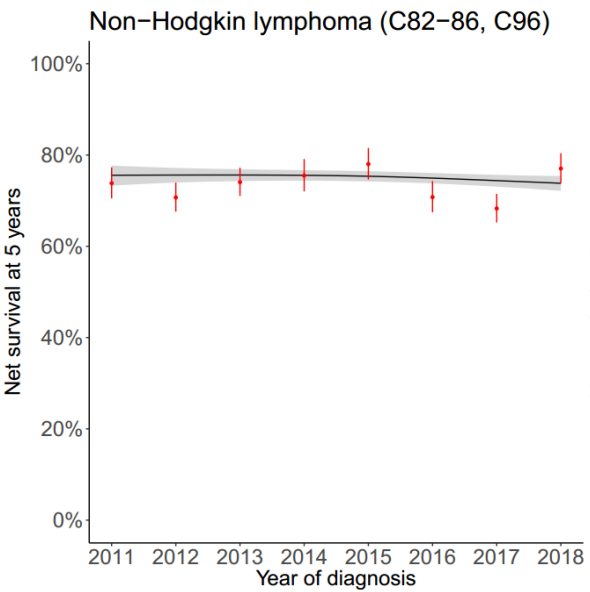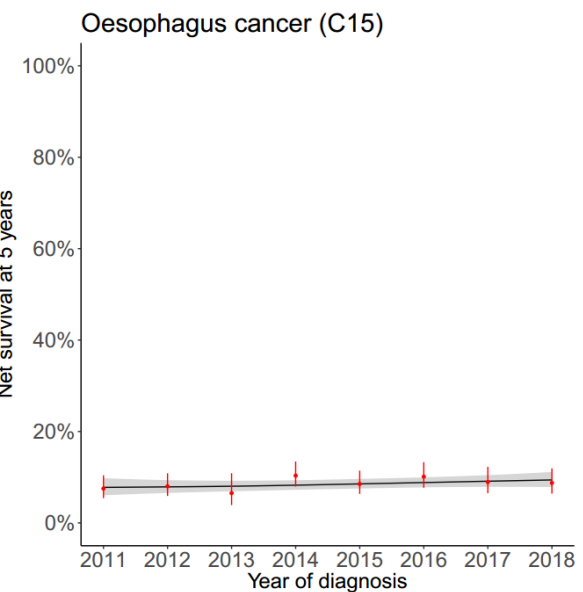

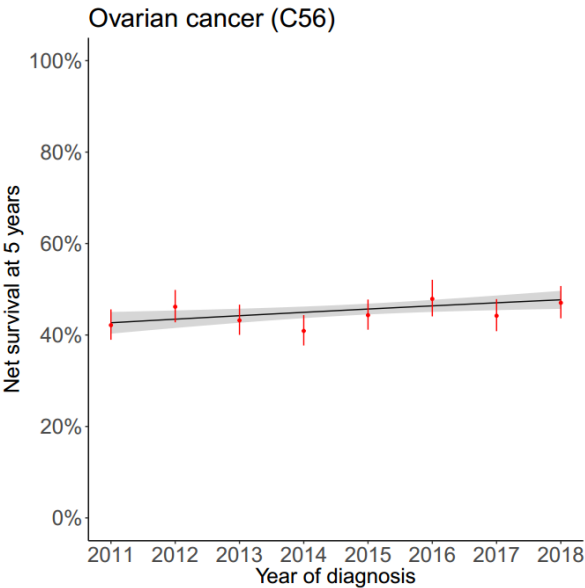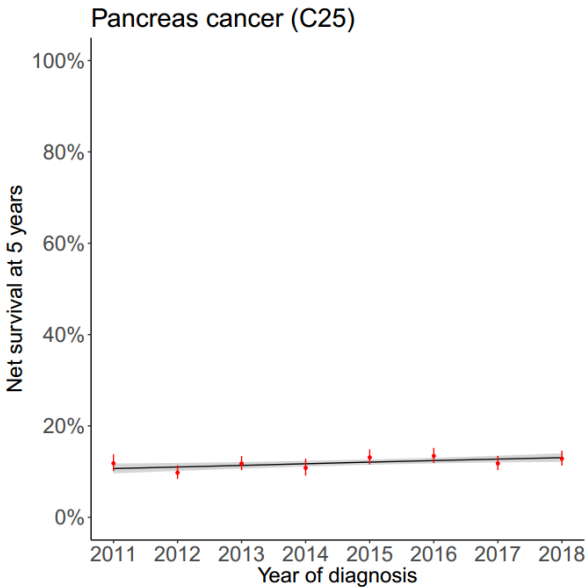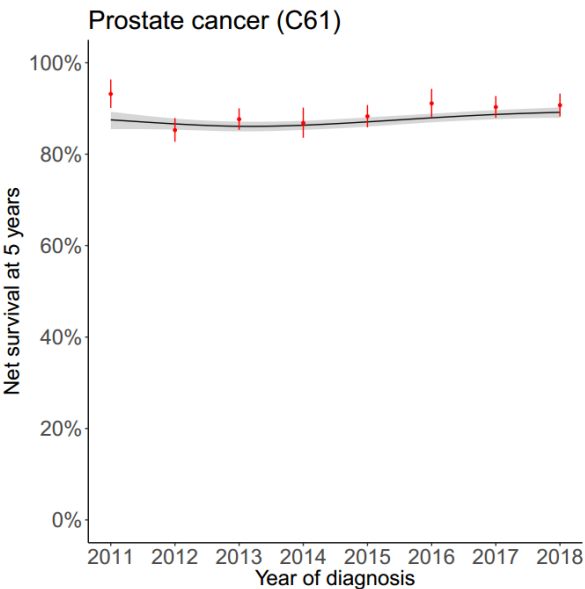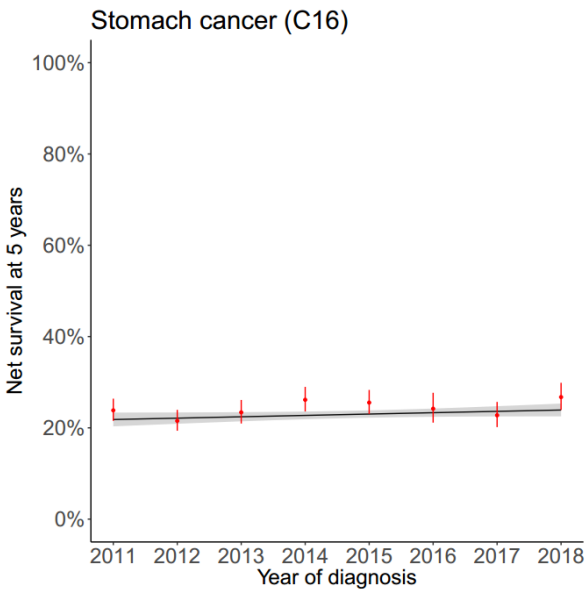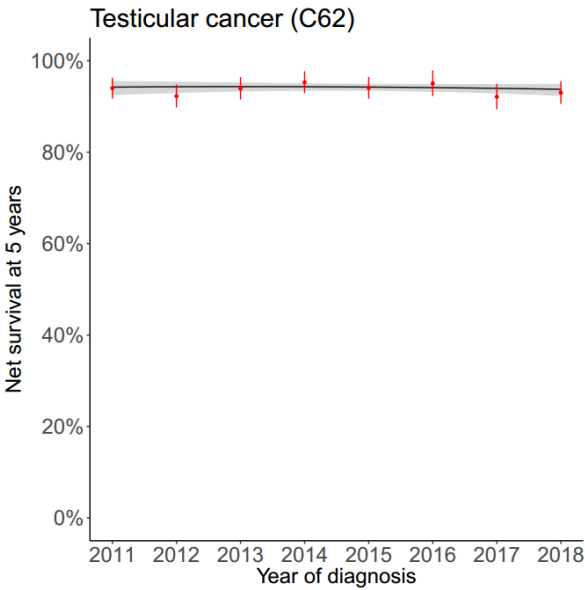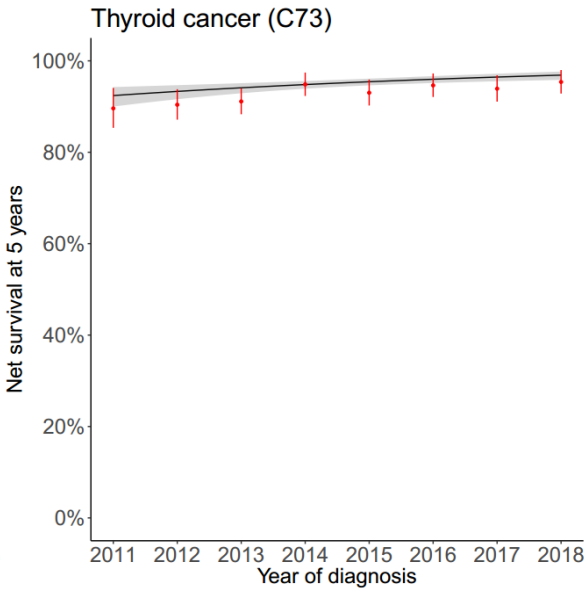

Supplement: Supplementary file 8 [file DataSheet1.pdf]
